# An Anti-proteome Nanobody Library Approach Yields a Specific Immunoassay for *Trypanosoma congolense* Diagnosis Targeting Glycosomal Aldolase

**DOI:** 10.1371/journal.pntd.0004420

**Published:** 2016-02-02

**Authors:** Steven Odongo, Yann G. J. Sterckx, Benoît Stijlemans, Davita Pillay, Théo Baltz, Serge Muyldermans, Stefan Magez

**Affiliations:** 1 Laboratory of Cellular and Molecular Immunology (CMIM), Vrije Universiteit Brussel, Brussels, Belgium; 2 Structural Biology Research Center, Vlaams Instituut voor Biotechnologie (VIB), Brussels, Belgium; 3 Department of Biotechnical and Diagnostic Sciences, College of Veterinary Medicine, Animal Resources and Bio-security (COVAB), Makerere University, Kampala, Uganda; 4 Laboratory of Myeloid Cell Immunology, VIB Inflammation Research Center, Ghent, Belgium; 5 Microbiologie Fondamentale et Pathogénicité, Centre National de la Recherche Scientifique, Université Bordeaux, Bordeaux, France; Institut Pasteur de Montevideo, URUGUAY

## Abstract

**Background:**

Infectious diseases pose a severe worldwide threat to human and livestock health. While early diagnosis could enable prompt preventive interventions, the majority of diseases are found in rural settings where basic laboratory facilities are scarce. Under such field conditions, point-of-care immunoassays provide an appropriate solution for rapid and reliable diagnosis. The limiting steps in the development of the assay are the identification of a suitable target antigen and the selection of appropriate high affinity capture and detection antibodies. To meet these challenges, we describe the development of a Nanobody (Nb)-based antigen detection assay generated from a Nb library directed against the soluble proteome of an infectious agent. In this study, *Trypanosoma congolense* was chosen as a model system.

**Methodology/Principal Findings:**

An alpaca was vaccinated with whole-parasite soluble proteome to generate a Nb library from which the most potent *T*. *congolense* specific Nb sandwich immunoassay (Nb474H-Nb474B) was selected. First, the Nb474-homologous sandwich ELISA (Nb474-ELISA) was shown to detect experimental infections with high Positive Predictive Value (98%), Sensitivity (87%) and Specificity (94%). Second, it was demonstrated under experimental conditions that the assay serves as test-of-cure after Berenil treatment. Finally, this assay allowed target antigen identification. The latter was independently purified through immuno-capturing from (i) *T*. *congolense* soluble proteome, (ii) *T*. *congolense* secretome preparation and (iii) sera of *T*. *congolense* infected mice. Subsequent mass spectrometry analysis identified the target as *T*. *congolense* glycosomal aldolase.

**Conclusions/Significance:**

The results show that glycosomal aldolase is a candidate biomarker for active *T*. *congolense* infections. In addition, and by proof-of-principle, the data demonstrate that the Nb strategy devised here offers a unique approach to both diagnostic development and target discovery that could be widely applied to other infectious diseases.

## Author Summary

The lack of diagnostic tests that are sensitive, affordable and user-friendly is the impediment to early detection and containment of infectious diseases. For these reasons, African trypanosomosis continues to pose serious threat to the communities that are unable to access laboratory services. Assays that are able to address above-mentioned challenges would greatly reduce the disease burden. Although few relatively sensitive agglutination assays for some forms of African trypanosomosis have been adapted to field conditions, the tests are not reliable indicators of active infections given the fact that they only detect antibodies. The barrier in the development of alternative tests capable of revealing ongoing infections through antigen detection is the lack of potent monoclonal antibodies that can compete with infection-induced host antibodies for the circulating parasite antigens. Using *T*. *congolense* as a model system, we demonstrate that Nanobodies (Nbs) targeting the parasite glycosomal aldolase can detect active infections. The strategy described addresses the technical shortcoming of conventional monoclonal antibody-based assay development by adopting an unbiased proteome screening approach combined with a phage panning strategy that is adapted to avoid interference of the infection-induced host antibody response. Hence, this study shows prospect for future development of Nb-based tests for other infectious diseases.

## Introduction

Infectious diseases are a leading cause of mortality and morbidity after non-communicable diseases worldwide [1]. Although the majority of these infectious diseases are treatable, the lack of better diagnostic facilities in the developing countries is a major impediment to their control [2]. While disease diagnosis based on clinical signs is relatively cheap, it is not reliable as some infections are latent, mixed, and/or cause pathologies with overlapping symptoms. In some cases, disease diagnosis based on clinical signs may be of little use as the symptoms only manifest themselves when the patient has entered a terminal stage. The diagnosis of infectious diseases is facilitated by the use of sophisticated molecular techniques such as PCR [2,3]. While these are reliable and increase the chance of detecting an infection even before the manifestation of clinical signs, their use in resource-constrained settings is untenable as they require automated equipment. To overcome the need for the latter, efforts are ongoing to adapt PCR to field conditions through point-of-care (POC) nucleic acid devices [4,5] and loop-mediated isothermal amplification [6]. The adoption of these POC nucleic acid techniques in developing countries remains uncertain given the fact that these new devices still operate on expensive reagents, require amplification steps, and sample processing. The majority of the cases of infectious diseases are found in developing countries [1,7], where basic laboratory facilities are scarce. Therefore, most laboratories in these parts of the world have resorted to relatively inexpensive (but less sensitive) microscopy-based techniques for routine diagnosis of infectious diseases. Among other factors, the over-reliance on microscopy contributes to the high burden of infections in these regions, as most pathogens escape detection thereby allowing their proliferation and continued transmission. Hence, there is an urgent need for diagnostic tools that can immediately and reliably detect active infections under field conditions.

Where nucleic acid-based assays are unaffordable and sensitivity of microscopy becomes limited, enzyme-linked immunosorbent assay (ELISA) can be used as an alternative tool for detection of infectious diseases through antibody [8] or antigen [9] detection. ELISA is a robust technique which operates without sophisticated equipments therefore favoring its application in less well-established laboratories such as those found in developing countries. Furthermore, ELISA can be easily adapted to non-laboratory conditions as exemplified by the availability of several immuno-based POC tests for important infectious diseases [10]. Antibody-based ELISA relies on the host immune system to amplify the detection limit. Two issues arise that need to be taken into account, i.e. reduced specificity due to cross-reactivity [11] and the inability to differentiate past from ongoing infections because of the persistence of antibodies in the circulation after clearance of infection [12,13]. While both issues are obviously avoided using an antigen detection format, the development of the latter faces its own hurdles. First, the antigen should be present in detectable quantities during an active infection [14] and should not cross-react with diagnostic tests used for the detection of other endemic parasites. Second, the capture and detection antibodies of the ELISA should be able to out-compete the host anti-pathogen antibodies which usually form immune complexes with the circulating antigen [15]. Nanobody (Nb) technology [16] offers prospects for the identification of native parasite antigens [17] as well as the detection of pathogens [18,19] and these have rejuvenated hope for refining the developments of ELISA and immuno-based POC tests. Nbs are recombinant proteins derived from the gene coding for the antigen binding portion of heavy-chain only IgG antibodies [20]. Nbs possess a relatively high thermostability [21,22] and are thereby attractive for the development of immunodiagnostic tests that could be applicable in hot climate such as the sub-Saharan Africa. Moreover, Nbs could be used to overcome certain challenges faced by tests based on monoclonal antibodies (MAbs). It has been documented that host antibodies usually sequester parasite antigens [15]. Given that MAbs are conventional antibodies, the host IgG molecules might conceal the epitopes from the MAbs employed in the diagnostic test [23]. Nb-based diagnostics could circumvent this binding interference caused by the host’s antibody response. Because Nbs possess extensive CDR3 loops [24], this allows tight binding of epitopes distinct from those recognized by conventional antibodies [25]. In other words, Nbs should detect both free antigens as well as those bound by host antibodies, which would make Nb-based diagnostic tests rather attractive. The exploitation of the Nb technology might expedite the development of potent ELISA and immuno-based POC tests for the endemic tropical diseases.

Here, we describe the development of a Nb-based ELISA for the specific detection of active pathogen infections. *Trypanosoma congolense*, the most important causative agent of African Animal Trypanosomosis (AAT), was chosen as a model system for our studies. Besides vector control, the only way to curb the spread of *T*. *congolense* infections is by case detection followed by drug treatment. Unfortunately, current diagnostic tests are not very reliable or user-friendly under field conditions. The buffy coat technique (BCT) [26], which is a general microscopy-based test for trypanosomes, is limited by sensitivity in cases where there is low parasitemia such as in chronic infections or where sample examination is delayed. While sensitive and specific detection of *T*. *congolense* infections can be achieved through species specific [27] or pan-trypanosome [28–31] PCRs, these molecular assays are not widely used for diagnosis of animal trypanosomes. Attempts to develop antibody-based ELISAs failed due to lack of specificity [32,33]. Even if it would have succeeded, the persistence of antibodies in the circulation several months after clearance of infections would compromise its application for establishing cure as was observed with the test for fascioliasis [12]. An attempt to develop an antigen detection ELISA test for *T*. *congolense* based on MAbs was hampered by a lack of sensitivity [34]. Thus, limitations of the current diagnostic tests for trypanosomes require that more research be done to provide assays that meet the expectations of end-users.

In this study, a potent Nb (Nb474) was obtained from an immune cDNA library prepared starting from the immunization of an alpaca with *T*. *congolense* TC13 soluble proteome. Nb474 was used for development of a *T*. *congolense* specific antigen detection homologous sandwich ELISA (Nb474-ELISA). The results presented in this study demonstrate the potential of Nbs as tools for the specific diagnosis of *T*. *congolense* infections and of *T*. *congolense* glycosomal fructose-1,6-bisphosphate aldolase (*Tco*ALD) as a candidate biomarker. Moreover, we show that the Nb474-ELISA is able to detect active *T*. *congolense* infections under experimental and natural conditions, and that it serves as a test-of-cure in experimental settings.

## Materials and Methods

### Ethical statement

All the animal experiments were performed according to directive 2010/63/EU of the European parliament for the protection of animals used for scientific purposes and approved by the Ethical Committee for Animal Experiments of the Vrije Universiteit Brussel (clearance numbers 11-220-6 and 13-220-3).

### Trypanosomes

The trypanosomes used in the study were obtained from various sources. *T*. *congolense* TC13 was kindly provided by Dr. Henry Tabel (Canada), *T*. *congolense* IL3000 was obtained from the laboratory of Microbiologie Fondamentale et Pathogénicité, Université de (Bordeaux), and *T*. *congolense* (STIB68, IL1180, TRT55, J423, Ruko14, MF3cl2, MF5cl4, Alick339c2 and Kapeya357c1) were obtained from the Institute of Tropical Medicine (Antwerp). *T*. *b*. *brucei* AnTat 1.1, *T*. *vivax* ILRAD700 and *T*. *evansi* STIB816 were also obtained from the Institute of Tropical Medicine (Antwerp).

### Animals, sample collection and examination

Mice (C57BL/6) approximately 6 weeks old (Janvier) were infected with 5000 trypanosomes per animal through intra-peritoneal route. Blood was collected by cardiac puncture (for one time bleeding) or peri-orbital route (for multiple collections at different time points). The blood was stored at 4°C for at least 17 h to allow clotting prior to serum collection for immediate examination or storage at -20°C. For quantification of parasites, the mouse tail tip was nipped with scissors and 2.5 μl of blood was drawn by pipette. The blood was diluted (1/200) in PBS and trypanosomes were counted using a Neubauer haemocytometer on a Nikon Eclipse TS 100 inverted microscope (Japan).

Cattle sera consisting of *T*. *congolense* IL1180 experimentally infected (n = 45) and negative control (n = 17) used for the evaluation of the Nb-ELISA were kindly provided by Prof. Luis Neves of the Faculty of Veterinary Medicine, Universidade Eduardo Mondlane (Mozambique). Hemolysed blood samples collected from domestic animals naturally infected with various endemic haemoparasites that were used to assess the cross-reactivity of the assay were kindly provided by Susan Ndyanabo of the Japanese International Cooperation Agency diagnostic laboratory, COVAB. The clinical samples comprised *T*. *congolense* positive cattle (n = 2), *T*. *brucei* positive cattle (n = 3) and dog (n = 2), *T*. *evansi* positive camel (n = 1), *T*. *theileri* positive cattle (n = 12), *Theileri parva* positive cattle (n = 44), *Babesia bigemina* positive cattle (n = 1), *B*. *canis* positive dog (n = 1), *Anaplasma marginale* cattle (n = 2) and goat (n = 2). The clinical samples were examined for trypanosomes by BCT and 18S-PCR-RFLP [30]. All the samples that were positive for *T*. *brucei* by 18S-PCR-RFLP were cross-checked for *T*. *evansi* by RoTat PCR [35] and non-RoTat PCR [36]. *T*. *parva*, *Babesia* sp and *A*. *marginale* were detected by Giemsa-stained smear.

### Propagation of trypanosomes, purification, and preparation of soluble proteome

*T*. *congolense* IL3000 BSF and PCF parasites were cultured and harvested as described [37]. The trypanosomes grown in mice were purified according to [38]. Briefly, infected blood was centrifuged [805 g, 10 min., 22°C], buffy coat was collected and loaded onto a PD-10 column (GE Healthcare) packed with Phosphate Saline Glucose pre-equilibrated DE-52 resin (Whatman) at pH 7.5 for purification of *T*. *congolense* and *T*. *vivax*; and pH 8.0 for purification of *T*. *b*. *brucei* and *T*. *evansi*. Trypanosomes eluted from the column were harvested by centrifugation (1811 g, 15 min. 22°C). The cell pellet was resuspended in 100 mM phosphate buffer pH 7.0 containing complete Protease Inhibitor Cocktail (Roche). Then, trypanosome soluble protein was prepared as described [39] and the protein concentration was measured by NanoDrop.

### Immunization of alpaca and Nb library construction

An alpaca was injected once a week, for six weeks, with 100 μg *T*. *congolense* TC13 soluble proteome potentiated with GERBU adjuvant LQ (GERBU BIOTECHNIK GmbH). On the 7^th^ week, 50 ml of blood was collected and peripheral blood lymphocytes were isolated for RNA extraction. cDNA was synthesized and amplified using Call001 and Call002 primers [39]. The amplicon was resolved on 1% agarose gel (Lonza) and eluted from the gel using GenElute Gel extraction Kit (Sigma) following the instructions of the manufacturer. The eluted DNA was amplified with nested primers A4short forward (5'-GATGTGCAGCTGCAGGAGTCTGGA/GGGAGG-3') and 38 reverse (5'-GGACTAGTGCGGCCGCTGGAGACGGTGACCTGGGT-3') followed by cloning in pHEN4 vector [40]. The library construction was ended with transformation of the ligated pHEN4 construct into fresh electrocompetent *Escherichia coli* (TG1) cells.

### Library panning and screening

For panning, the library was transfected with 10^12^ M13K07 phage particles (Invitrogen) to obtain display phage library. Then 10^11^of display phages were adsorbed on a 96-well Nunc plate (Thermo scientific) coated in parallel with soluble proteome (25 μg/well) of four different *T*. *congolense* strains (STIB68, TRT17, TC13 or MF3cl2). Bound phages were eluted with 100mM triethylamine (pH~10) and neutralized with 100 μl 1M Tris (pH 8.2). After three panning cycles, enrichment was assessed by phage ELISA. For phage ELISA, soluble proteins of the four *T*. *congolense* strains used in panning were coated in parallel on Nunc plate (1μg/well) and uncoated (control) wells were filled with PBS (100 μl). Display phages (10^10^) of panning rounds 0–3 were added to coated and uncoated wells. The fifth pair (blank) of wells were filled with blocking buffer only. Thereafter, anti-M13 conjugate horse radish peroxidase (HRP) (100 μl) (GE Healthcare) diluted (1/2000) in blocking buffer was added followed by 1-Step ultra 3,3′,5,5′-tetramethylbenzidine (TMB) substrate (Thermo scientific) added at (100 μl/well). The reaction was stopped with 1M sulphuric acid (50 μl/well). OD_450nm_ was read on spectrophotometer (EL_X_808 Ultra-microplate reader, Bio-Tek instruments) using Gen5 software. For screening, single colonies (of the panning rounds 2 and 3) were expressed in Terrific Broth medium as mini culture (1ml) supplemented with ampicillin and 1 mM isopropyl-β-D-thiogalactosidase (IPTG). Cells were harvested, disrupted by osmotic shock and the periplasmic extracts (100 μl) were added to wells coated with soluble proteome (1 μg/well). A screening strategy was adopted in order to maximize chances of selecting *T*. *congolense* cross-reactive Nbs. Thus, clones obtained after panning on TC13 soluble proteome were screened on MF3cl2 soluble protein; clones obtained after panning on MF3cl2 soluble proteome were screened on TC13 soluble proteome; clones obtained after panning on STIB68 soluble proteome were screened on TRT17 soluble proteome; and clones obtained after panning on TRT17 soluble proteome were screened on STIB68 soluble proteome. Negative control wells (also coated with soluble proteome) were filled with PBS (100 μl) instead of periplasmic extract. Positive binders were detected using biotinylated mouse Anti-HA IgG (Eurogentec), HRP-conjugated streptavidin (strep-HRP) (Jackson ImmunoResearch laboratories) and TMB substrate. Colonies were considered positive when the ratio of OD between the test and control wells was ≥ 2. All the positive binders selected were re-screened on naive mouse serum diluted (1/10) in PBS in order to identify mouse protein binders. Thereafter the genes of *T*. *congolense* specific binders were sequenced and the data was processed using Clone Manager 9 Professional Edition software employing the BLASTN 2.2.29+ program [41].

### Re-cloning the Nb gene in expression vectors, protein production and purification

The Nb gene in pHEN4 construct was amplified by PCR followed by double digestion with *Pst*I and *Eco*91I. The fragment was ligated into pHEN6c as well as pBAD expression vectors for incorporation of a hexahistidine tag (His_6_-tag) and biotin acceptor domain (for *in vivo* biotinylation), respectively. Whereas the Nb ligated pHEN6c construct was transformed alone in *E*. *coli* WK6, the ligated pBAD construct was co-transformed with BirA plasmid which aid *in vivo* biotinylation. For expression, starter culture (1 ml) was inoculated in 330 ml terrific broth (TB) supplemented with ampicillin (100 μg/ml) for cells transformed with pHEN6c or ampicillin (100 μg/ml) and chloramphenicol (35 μg/ml) for cells co-transformed with pBAD/BirA. Cells were grown at 37°C until an OD_600nm_ of ± 0.6 and expression induced with 1 mM IPTG. For culture expressing biotinylated Nb, 0.05 mM D-biotin (ACROS Organics) was added 30 min before induction. After induction, cells were grown overnight at 28°C. The next morning cells were harvested and lysed by osmotic shock. The His_6_-tag Nb (Nb474H) and biotinylated Nb (Nb474B) in the perisplasmic extract was affinity purified on His-Select Nickel (Sigma) and streptavidin mutein matrix (Roche), respectively, followed by size exclusion chromatography on ÄKTA (GE healthcare). Protein production was analyzed by SDS-PAGE under reducing conditions using 10% Bis-Tris gel (Novex Life Technologies) and Western blot.

### Generation of the Nb474-ELISA protocol

The aim of this study was to develop a Nb-based sandwich test such as described previously [42]. Therefore, the two Nb formats (Nb474H and Nb474B) were titrated in a checkerboard fashion in order to arrive at optimal concentration of capturing and detection Nbs required for the highest signal intensity (OD_450nm_). In this experiment, the capturing Nb (Nb474H*)* was titrated against detecting Nb (Nb474B) using *T*. *congolense* TC13 soluble protein (S1 Materials and Methods). After checkerboard titration, a protocol for Nb474-based homologous sandwich ELISA was established whereby Nunc plate was coated with 100 μl/well Nb474H (0.02 μg/ml) in PBS and incubated overnight at 4°C. The following morning, the non-coated Nb was discarded and wells washed with PBS-T three times. Washed wells were blocked with 300 μl 5% milk for 2 h at 22°C. The blocking buffer was discarded and wells were washed three times. Test samples were added (100 μl/well) and incubated for 1 h at 22°C. Wells were emptied and washed three times. Then, Nb474B (100 μl) diluted to 0.02 μg/ml was added per well and incubated for 1h at 22°C. Wells were emptied and washed four times. Strep-HRP diluted to 1 μg/ml in blocking buffer was added (100 μl/well) followed by incubation for 1 h at 22°C. Conjugate was discarded, wells were washed five times and TMB substrate was added at 100μl/well. Color development allowed to progress for at least 25 min and the reaction was stopped using 1M sulphuric acid (50 μl/well) and OD_450nm_ was read.

### Detection limit of Nb474-ELISA

To determine the detection limit of the Nb474-ELISA, *T*. *congolense* TC13 infected mouse blood (with 5.86x10^8^ trypanosomes/ml) was serially diluted (4-fold) in naive mouse blood. The set of samples were incubated overnight at 4°C. The following day, plasma was harvested and then tested by ELISA.

### Establishment of the ELISA performance upon prolonged storage of infected sera

To test the ELISA upon prolonged storage at different temperatures, mice infected with *T*. *congolense* (n = 10) were bled on day 7 post-infection. Sera were collected and pooled followed by incubation at different temperature (4, 22 or 37°C) for a specified number of days (1, 3, 5 or 7). The sera aliquots were tested by Nb474-based ELISA and the OD_450nm_ was compared.

### Test-of-cure assay

To assess if the Nb474-ELISA can be used as a tool for monitoring cure, an infection-treatment assay was performed and OD_450nm_ was monitored alongside blood parasite load. In this experiment, mice were randomly assigned to two groups of 6 animals each. On day 0 post-infection, baseline sera were obtained from all the subjects and pooled by group. Thereafter, all the subjects in each group were infected with *T*. *congolense* TC13. On day 7, group 2 was treated with 0.8 mg Berenil in 50 μl distilled water. During the course of the experiment (from day 3 until day 21) mice were bled on alternate days in order to monitor the level of antigen and the concentration of trypanosomes in the circulation. In addition to microscopy, cure of treated mice was assessed during four weeks by sub-inoculation of naïve mice with blood collected from the treated group on weekly basis.

### Labeling Nb474 and antigen localization by immunofluorescence analysis

Nb474 was labeled at the lysine residues with Alexa Fluor 488 (Invitrogen Molecular probes) following the instructions of the manufacturer. After labeling, the Nb (0.3 μg) was incubated with purified motile as well as paraformaldehyde fixed *T*. *congolense* BSF cells and no binding was detected. Thereafter, the Nb (0.3 μg) was incubated with cells that were fixed and permeabilized using a commercial kit (BD Biosciences) following the instructions of the manufacturer followed by incubation with 1 mg/ml 4’,6’-diamidino-2-phenylindle (DAPI) for 5 min. The preparation was examined using a Zeiss Axio Imager Z1 fluorescence microscope. Based on binding result obtained from the second experiment, co-localization of Alexa Fluor 488 labeled Nb474 with a *Tco*ALD cross-reactive anti *T*. *brucei* aldolase (*Tb*ALD) monoclonal antibody (Anti-*Tb*ALD MAb) (ProteoGenix) against synthetic peptide PEVMIDGTHDIETCQRVSQHVWS was performed on cultured *T*. *congolense* IL3000 bloodstream form (BSF) and procyclic form (PCF) parasites. In this experiment, the cultured cells were resuspended in 320 μl 1% (v/v) formaldehyde in PBS for 10 min. Then 1M glycine dissolved in PBS (40 μl) was added and mixed by a pipette. The mixture was incubated for 10 min and diluted with PSG (800 μl). The dilution (20 μl) was placed in different wells on a microscope slide (Thermo scientific) and the liquid on the slide was dried in a fume hood. Wells were washed three times with 50 μl BSA (1 mg/ml) in PBS. An Anti-*Tb*ALD MAb (20 μl) diluted (1/10) in a diluent solution [1mg/ml BSA in PBS containing 0.1% (v/v) Triton X-100] was added to the wells and incubated for 1 h. Wells were washed three times. Alexa Fluor 594 labelled goat anti-mouse (Invitrogen) (20 μl) diluted (1/100) in a diluent solution containing Alexa Nb474 (3 μg/ml) was added to the washed slide. The slide was then kept in a dark humidified box for 45 min and washed with PBS (50 μl/well) three times. DAPI was added to the washed slide and kept in the dark for 3 min. Wells were washed with PBS (50 μl/well) three times and the preparation was examined immediately.

### Affinity purification of the target antigen

Starting materials for the purification of the antigen from soluble proteome and plasma were 7 mg *T*. *congolense* soluble proteome diluted in 20 ml PBS and 30 ml of serum collected at the first peak parasitemia. The purification process was started by concentrating the samples using a 100,000 molecular weight cut-off (MWCO) concentrator (Sartorius stedim biotech). Thereafter both fractions (the concentrate and filtrate) were tested by Nb-ELISA after normalization for protein concentration. A higher OD_450nm_ was recorded in the concentrate compared to filtrate. Then, the concentrate was washed twice by adding 5ml PBS to the concentrator and draining by centrifugation (453 g, 4°C) until 1 ml. Washed concentrate was stored at -20°C until the antigen was purified.

The homologous Nb sandwich ELISA was then employed to isolate the native target antigen from the *T*. *congolense* soluble proteome, the sera collected from *T*. *congolense* infected mice, and the secretome of *T*. *congolense* IL3000. Four Nunc plates were coated with capturing Nb (Nb474H) (5 μg/well) overnight at 4°C. The following morning, coating was discarded and the plate was washed three times. Protein-Free T20 Blocking Buffer (Thermo scientific) was added (300 μl/well) for 3 h at 22°C. Blocking buffer was discarded and wells were washed three times. Soluble proteome diluted in blocking buffer was added (10 μg/well) to the first pair of the plates and the second pair of the plates was filled with sera (100 μl/well) diluted (1/10) in blocking buffer. Binding of the target antigen to Nb474H proceeded for 2 h at 22°C and unbound molecules were discarded. Detection Nb (Nb474B) was added (2 ng/well) and incubated for 1 h at 22°C. Subsequently, the unbound Nb474B was discarded and the wells were washed four times. Strep-HRP was added to four control wells that were purposely included to serve as reporter for sandwich formation. The rest of the wells were filled with blocking buffer (100 μl/well) for 1 h at 22°C. The plates were emptied and washed five times. Control wells were filled with TMB (100 μl/well) and wells where captured antigen was to be eluted were filled with 0.3M glycine HCl pH 3.0 (100μl/well). Elution was proceeded with gentle rocking on bench top shaker over 30 min at 22°C. The eluted samples were collected and pooled according to the starting material (soluble proteome or sera). Solutions obtained from each pair of plates (about 19.2 ml) was concentrated to 1 ml using a 5000 MWCO concentrator of 15 ml capacity and then to 30 μl using a 3000 MWCO concentrator of 400 μl capacity. The concentrators with low MWCO and capacities were employed in the last steps in order to trap the eluted native target antigen-Nb complex as well as dissociated Nbs. The eluted samples (6 μg from lysate plates and 8 μg from serum plates) were resolved on SDS-PAGE alongside Nbs (Nb474H and Nb474B) as control and proteins visualized using silver staining [43]. The purification of the antigen from secretome was performed by QuickPick IMAC (Bio-Nobile) following the instructions of the manufacturer. Subsequently, Nb474H, flow through, wash, and eluted samples were resolved on SDS-PAGE under reducing conditions followed by staining with coomassie blue. The proteins in the bands at ± 40 kDa that appeared in all the three eluted samples were identified by mass spectrometry.

### Mass spectrometry identification of *T*. *congolense* glycosomal aldolase

Bands of interest were excised from the SDS-PAGE gel, digested with trypsin, and analyzed by liquid chromatographic tandem MS as described [44]. Briefly peptides were separated by an acetonitrile gradient on a C18 column and the MS scan routine was set to analyze by MS/MS the five most intense ions of each full MS scan, dynamic exclusion was enabled to assure detection of co-eluting peptides.

Protein identification was performed with SequestHT. In details, peak lists were generated using extract-msn (ThermoScientific) within Proteome Discoverer 1.4.1. From raw files, MS/MS spectra were exported with the following settings: peptide mass range: 350–5000 Da, minimal total ion intensity 500. The resulting peak lists were searched using SequestHT against a target-decoy *T*. *congolense* protein database (33144 entries comprising forward and reversed sequences) obtained from the Welcome Trust Sanger Institute (http://www.sanger.ac.uk/resources/downloads/protozoa/trypanosoma-congolense.html). The following parameters were used: trypsin was selected with proteolytic cleavage only after arginine and lysine, number of internal cleavage sites was set to 1, mass tolerance for precursors and fragment ions was 1.0 Da, considered dynamic modifications were +15.99 Da for oxidized methionine. Peptide matches were filtered using the q-value and Posterior Error Probability calculated by the Percolator algorithm ensuring an estimated false positive rate below 5%. The filtered Sequest HT output files for each peptide were grouped according to the protein from which they were derived and their individual number of peptide spectral matches was taken as an indicator of protein abundance. The raw MS data were then searched against a *T*. *brucei* /*T*. *congolense* database.

### Nb474H and anti-*T*. *brucei* aldolase MAb heterologous sandwich ELISA

Of the proteins identified by mass spectrometry, *Tco*ALD had the highest score. Thus, in order to establish if Nb474 was indeed binding to glycosomal aldolase, formation of a sandwich between the Nb474H and Anti-*Tb*ALD MAb was assessed in a heterologous sandwich ELISA where *T*. *congolense* TC13 soluble proteome was used as the antigen source. In this ELISA, a 96-well Nunc plate was coated (5 μg/well) with Nb474H diluted in PBS overnight at 4°C. The following morning, coated sample was discarded; wells were washed three times and blocked with 300 μl 5% milk for 2 h at 22°C. Blocking was discarded and wells washed three times. The soluble proteome diluted (100 μg/ml) in blocking buffer was added to the plate (100 μl/well) and incubated for 1 h at 22°C. Wells were emptied and washed three times. An Anti-*Tb*ALD MAb diluted (1/100) in blocking buffer was added (100 μl/well) to the plate and incubated for 1 h at 22°C. The antibody was discarded and wells washed three times. Anti-mouse HRP conjugate diluted (1/1000) in blocking buffer was added (100 μl/well) and incubated for 1 h at 22°C. The conjugate was discarded and wells were washed five times. The ELISA was developed and stopped as described earlier.

### Cloning glycosomal aldolase genes, expression and Nb474 binding analysis

Nucleotide sequences coding for aldolase of *T*. *congolense* (EMBL-EBI accession no. CCC93713.1), *T*. *b*. *brucei* (EMBL-EBI accession no. M19994.1) and *Leishmania mexicana* (EMBL-EBI accession no. CAB55315.1) were retrieved from the GenBank and codon-optimized for expression in *E*. *coli*. The codon-optimized sequences were further processed by incorporating nucleotides coding for His_6_-tag and a proximal tomato etch virus cleavage site. Finally, *Nde*I and *Xho*I cutting sites were added to the 5′ and 3′, respectively, followed by *in silico* construction in pET-21b (+) expression vector. Assembled sequences were submitted to a commercial company (GenScript) for synthesis and ligation in the expression vector. The constructs were electro-transformed into *E*. *coli* BL21(DE3) and glycerol stocks of the transformed cells were stored at -80°C. The expression of recombinant aldolase was performed as previously described [45]. During the expression, aliquots (5 ml) of the culture were taken 0 h and 18 h post-induction for preparing crude soluble protein. The aliquots were harvested by centrifugation (11,325 g, 8 min., 4°C) and the pellet was completely resuspended in lysis buffer (50 mM Tris pH 7.0, 500 mM NaCl) for 1 h at 4°C followed by sonication while on ice. The crude lysate protein concentration was measured by NanoDrop and expression was checked by SDS-PAGE as well as Western blot. Since Nb474 could not detect aldolase expression in Western blot, ELISA was employed for this purpose whereby the crude soluble proteins (10 μg/well) were probed with homologous (Nb474H-Nb474B) as well as heterologous (Nb474H-Anti-*Tb*ALD MAb) sandwich ELISA. Concurrently, ELISA involving Anti-His IgG and Anti-*Tb*ALD MAb were performed on crude soluble proteins that were coated (10 μg/well) as positive controls.

### Statistical analysis

Unpaired *t*-tests used for group comparisons and the performance of the Nb-based ELISA (sensitivity and specificity) were analyzed by GraphPad Prism (version 6.03). Values of *p* ≥ 0.05 were considered non-significant (ns). Where *, **, *** and ****denote *p*<0.05, *p*<0.01, *p*<0.001, and *p*<0.0001, respectively.

### Accession numbers

The sequences of *T*. *congolense*, *T*. *b*. *brucei* and *L*. *mexicana* aldolase genes are available from EMBL-EBI nucleotide sequence database with accession numbers CCC93713.1, M19994.1 and CAB55315.1, respectively.

## Results

### Identification of a *T*. *congolense* specific binder (Nb474) from an anti-*T*. *congolense* soluble proteome Nb library

**Fig 1:**
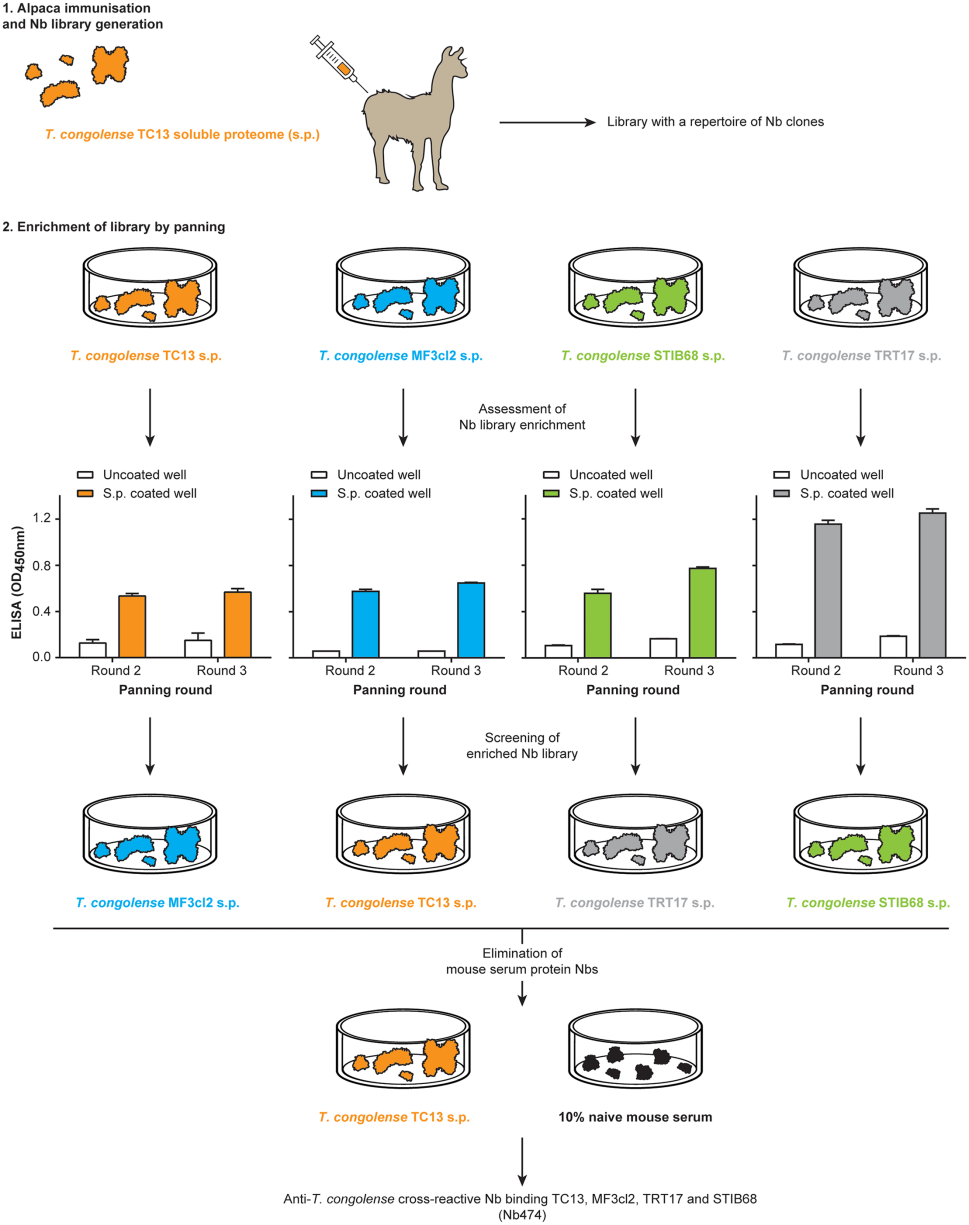
Schematic illustration of the Nb library generation, panning and screening strategy. The Nb library was generated from an alpaca that was immunized with *T*. *congolense* TC13 soluble proteome (s.p.). Parallel panning was performed on s.p. of *T*. *congolense* homologous strain (TC13) or heterologous strains (MF3cl2, STIB68 and TRT17). Then panning round 2 and 3 were screened on heterologous strains for colonies expressing anti-*T*. *congolense* s.p. Nbs. All the positive colonies were re-screened on TC13 s.p. as well as mouse serum in order to identify and eliminate those expressing non-specific Nbs.

Using the approach summarized in Fig 1, the most potent *T*. *congolense* specific binder (Nb474) was obtained. The Nb library, generated against a *T*. *congolense* TC13 soluble proteome preparation, was panned in parallel on soluble proteomes prepared from various *T*. *congolense* strains (TC13, MF3cl2, STIB68 and TRT17) to obtain *T*. *congolense* cross-reactive binders. Each of the enriched libraries was screened on heterologous strains whereby 101 positive colonies were identified. The 7 cross-reactive binders interacting with mouse serum components were excluded in the procedure (S1 Fig). Thereafter, the remaining 94 clones were amplified and sequenced. All the in-frame sequences belonged to the same CDR3 sequence family (S2 Fig).

### Specific diagnosis of experimental *T*. *congolense* infections in mice using a Nb474-ELISA

**Fig 2:**
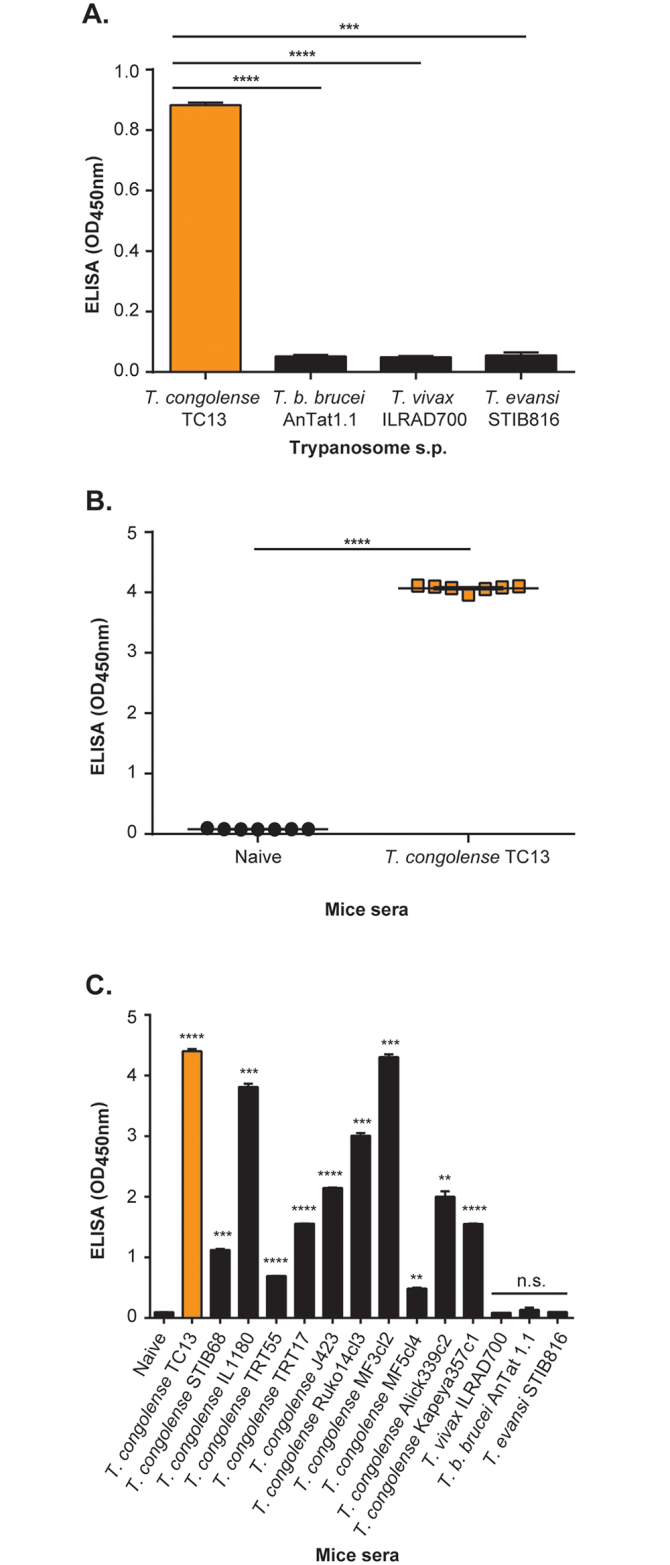
Specific recognition of *T*. *congolense* by the Nb474-ELISA. (A) ELISA binding assay performed on trypanosome soluble proteome (s.p.) reveals specific recognition of *T*. *congolense*. (B) ELISA binding assay performed on sera collected from naive as well as *T*. *congolense* infected mice shows the ability of the ELISA to detect infected animals. (C) Assessment of the ELISA on pooled sera collected from mice infected with different species of trypanosomes indicates specific detection and cross-reaction with various strains of *T*. *congolense*. The OD_450nm_ shown on the graph represent the average value recorded from the duplicate wells. Results are expressed +/- standard deviation (SD) and statistical analysis was performed by comparing the OD of non-infected animals with those of infected animals. n.s. = non-significant, ** p<0.01, *** p<0.001, **** p<0.0001.

The aim of this study was to develop an assay for the specific diagnosis of *T*. *congolense* infections. The assay consists of a Nb474-based homologous sandwich ELISA, in which a His-tagged version of the Nb was used for antigen capturing (Nb474H), while a biotin version of the Nb was used for detection (Nb474B). To test the cross-reactivity of the Nb474-ELISA, the latter was validated on soluble proteome of different species of animal infective trypanosomes. As presented in Fig 2A, a significant high optical density (OD_450nm_) was only recorded on *T*. *congolense* soluble proteome, whereas extracts from *T*. *b*. *brucei*, *T*. *vivax* or *T*. *evansi* score negative. Next, the ELISA was evaluated for its capacity to diagnose experimental *T*. *congolense* infections in mice. When comparing the sera of *T*. *congolense* positive mice to the sera of naive controls, a significant difference in the average OD_450nm_ of about 4.0 was observed between both groups (Fig 2B). Having established that the Nb474-ELISA was specific for *T*. *congolense* and that it can detect an active infection, the assay was validated in a set-up in which mice were infected with different strains of *T*. *congolense* and other species of trypanosomes. While the ELISA identifies *T*. *congolense* infected animals irrespective of the parasite strain, the test scores negative on mice infected with trypanosome species other than *T*. *congolense* (Fig 2C).

Additionally, the detection limit of the Nb474-based assay was also investigated as uneven OD scores on sera collected from various *T*. *congolense* strains are attributed to differences in parasite density. To establish the assay’s detection limit, a set of sera samples prepared from blood having different concentrations of *T*. *congolense* TC13 parasites were tested in the homologous Nb474 sandwich ELISA. The lowest parasite concentration yielding a signal above the threshold OD_450nm_ (at ≥2-fold negative sample) is 1.43x10^5^ trypanosomes/ml (S3 Fig).

Finally, to alleviate the fear of possible loss of sensitivity of the test in case examination of sample is delayed as often observed with BCT, the ELISA was evaluated on *T*. *congolense* infected sera samples that were incubated at different temperatures for specified time periods. The results illustrate that, storing samples at 4°C or 22°C for up to 7 days does not affect the ELISA signal. However, raising storage temperature to 37°C for 3, 5 and 7 days reduced the signal up to 2, 10 and 44 folds, respectively (S4 Fig). Given that the ELISA signal is unaffected by sample storage at 37°C within a relatively short time frame (i.e. 1 day), this would enable the applicability of the Nb474-ELISA under realistic ‘field conditions’ where prompt examination of samples is usually delayed by an entire day due to electricity failures or transport to diagnostic laboratories.

### Application of the Nb474-ELISA as a test-of-cure under experimental conditions

**Fig 3:**
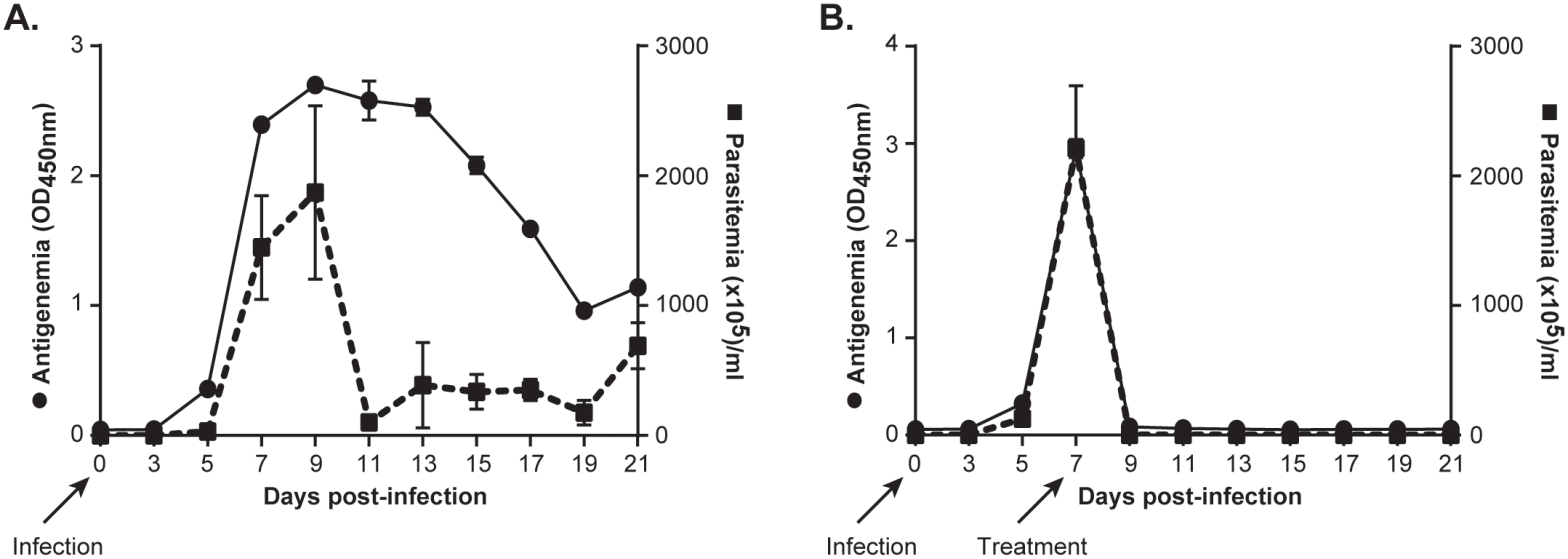
Effect of Berenil treatment intervention on antigenemia in mice. (A) The progression of antigenemia and parasitemia with no treatment intervention. Antigen levels constantly remained above baseline (naive state) when there is active infection. (B) The progression of antigenemia and parasitemia before and after Berenil treatment. Berenil treatment cleared circulating parasites causing a sharp decline in the levels of circulating antigen. The OD_450nm_ shown on the graph represent the average value recorded from pooled sera analyzed in duplicate. Results are representative of two experiments consisting of 5 mice per group and expressed as +/- standard deviation (SD). Arrows are showing time points where mice were infected with trypanosomes and treated with Berenil.

Next, the Nb474-ELISA was assessed for its capacity to differentiate animals with ongoing infections from those cured from trypanosomes after Berenil treatment in an experimental set-up. During the entire course of the experiment, the antigenemia and the parasitemia were monitored by the Nb474-ELISA and microscopy, respectively. During the early stages of the active infection the antigen profile correlates well with parasitemia (Fig 3A). After the first parasitemia peak, when the parasitemia is drastically reduced due to host immune intervention, the antigen level remains high for 4 days before showing a gradual decline. In contrast, after drug-mediated parasite clearance, no antigen could be detected (Fig 3B). Altogether, these results show that in experimental infections, the Nb474-ELISA can be used as a test-of-cure.

### Validation of the Nb474-ELISA for *T*. *congolense* diagnosis in cattle

**Fig 4:**
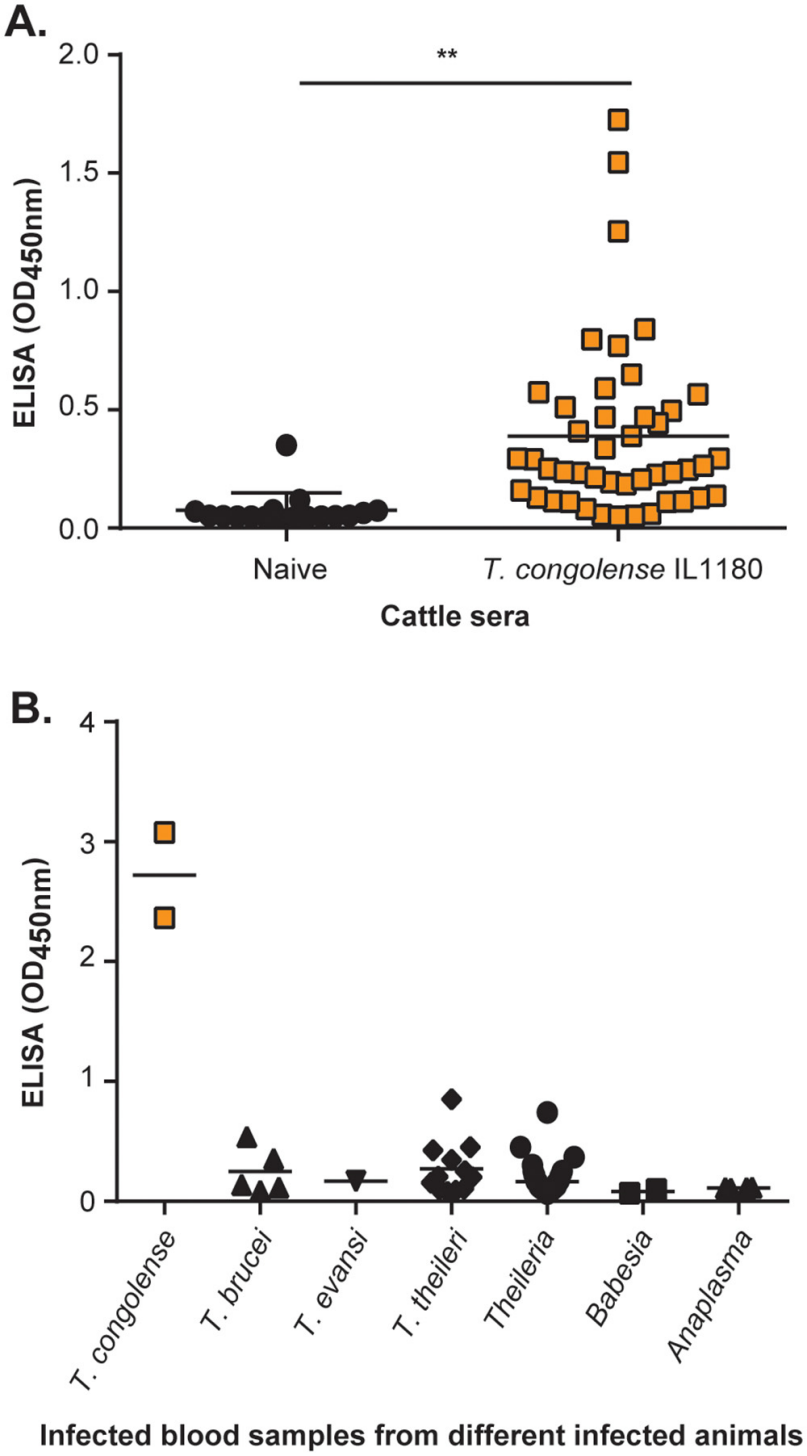
Nb474-ELISA detected *T*. *congolense* infections in experimentally and naturally infected cattle. (A) Examination of sera collected from naive and cattle experimentally infected with *T*. *congolense* IL1180. Average OD_450nm_ recorded on sera of naive cattle was significantly lower than those of infected cattle. (B) Examination of a panel of field blood samples collected from various domestic animals positive for different endemic tropical parasites has shown that the ELISA efficiently detected samples in two field samples collected from cattle that were naturally infected with *T*. *congolense*. The OD_450nm_ values plotted on the graph represents the average value recorded from the duplicate wells. ** p<0.01.

The diagnostic performance of the Nb474-ELISA for the specific identification of *T*. *congolense* infections in cattle was examined by testing sera collected from cattle experimentally infected with *T*. *congolense* IL1180 (n = 45) and *T*. *congolense* negative animals (n = 17). These results, of which the ELISA OD_450nm_ scores of the individual serum samples are displayed in Fig 4A, were employed to determine the i) Sensitivity (defined as the proportion of the infected cattle that scored positive), ii) Specificity (defined as the proportion of uninfected cattle that scored negative), iii) Positive Predictive Value (PPV; defined as the proportion of the true positive in those samples that scored positive) and iv) Negative Predictive Value (NPV; defined as the proportion of true negative in those sera that scored negative) of the Nb474-ELISA. The results have been summarized in Table 1. The test has a sensitivity and specificity of 87% [95% confidence interval (CI), 73% to 95%] and 94% [95% CI, 71% to 100%], respectively. The PPV is 98% [95% CI, 87% to 100%], whereas the NPV is 73% (95% CI, 50% to 89%).

**Table 1:** Performance of the Nb474-based ELISA on *T*. *congolense* IL1180 experimentally infected cattle and naive animals.

	Infection status of cattle sera		
Nb474-ELISA result	*T*. *congolense* IL1180 positive	Naive	Total
Positive	39^a^	1^b^	40
Negative	6^c^	16^d^	22
Total	45	17	62

Sensitivity = 39/(39+6), Specificity = 16/(1+16), Positive Predictive Value = 39/(39+1) and Negative Predictive Value = 16/(6+16).

^a^True positive,

^b^false positive,

^c^false negative,

^d^true negative

The Nb474-ELISA was then tested on clinical field samples to assess the ability of the Nb474-based assay to specifically detect natural *T*. *congolense* infections. All clinical field samples were investigated by other techniques (Giemsa-stained smear, BCT, PCR) to confirm infection with *T*. *congolense* and/or any other endemic tropical parasites. When applying the Nb474-ELISA, the OD_450nm_ observed on *T*. *congolense* positive samples is higher than on any other samples tested (Fig 4B), demonstrating that the ELISA can be used to specifically detect *T*. *congolense* infections in a natural setting.

### Immunofluorescence localization, capturing and identification of the Nb474 target

**Fig 5:**
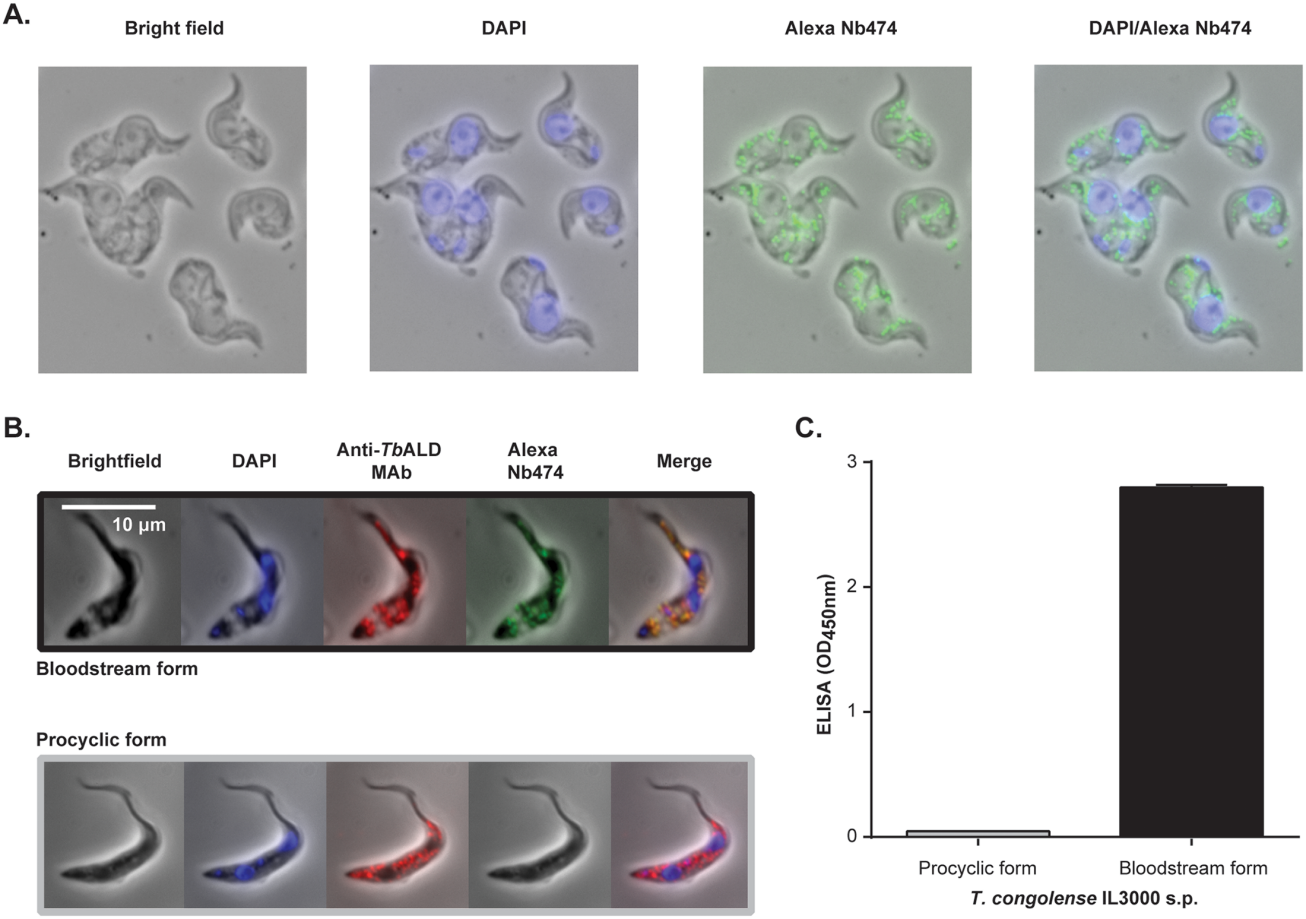
Immunofluorescence and ELISA revealed that Nb474 targets a protein in the *T*. *congolense* BSF. (A) Preliminary analysis of Alexa-labeled Nb474 binding to fixed and permeabilized *T*. *congolense* BSF parasites reveals green intra-cytoplasmic structures green (panels 3 and 4). (B) Co-localization of Alexa labeled Nb474 and anti-*T*. *brucei* aldolase MAb (Anti-*Tb*ALD) performed on *T*. *congolense* BSF shows the binding of the Nb matched with the Anti-*Tb*ALD. (C) The Nb474-ELISA specifically recognizes *T*. *congolense* BSF soluble proteome (s.p.), which is an indication of target uniqueness to the BSF.

To identify the target of Nb474, a localization study was performed whereby cultured *T*. *congolense* IL3000 BSF parasites were probed with Alexa Fluor labeled Nb474. The fluorescent intracellular structures observed (Fig 5A, panels 3 and 4) had the typical appearance of glycosomes [46,47]. To investigate the hypothesis that Nb474 targets a glycosomal component, a co-localization experiment was carried out using Alexa Fluor labeled Nb474 and an Anti-*Tb*ALD, which is a pan trypanosome marker for the glycosomes and recognizes a linear epitope. This was performed on both *T*. *congolense* BSF and PCF parasites. While a clear co-localization is observed for the BSF parasites, Nb474 does not label the PCF parasites (Fig 5B). To verify whether Nb474 indeed binds an antigen that is unique to BSF parasites, the Nb474-ELISA was used to probe the presence of the target antigen in a soluble proteome preparation obtained from both forms of the parasite. As it can be seen from Fig 5C, the ELISA scores positive for the BSF parasites, while no signal is observed for the PCF parasites. Collectively, these experiments demonstrate that Nb474 targets a glycosome-associated soluble protein that is specific to *T*. *congolense* BSF parasites.

**Fig 6:**
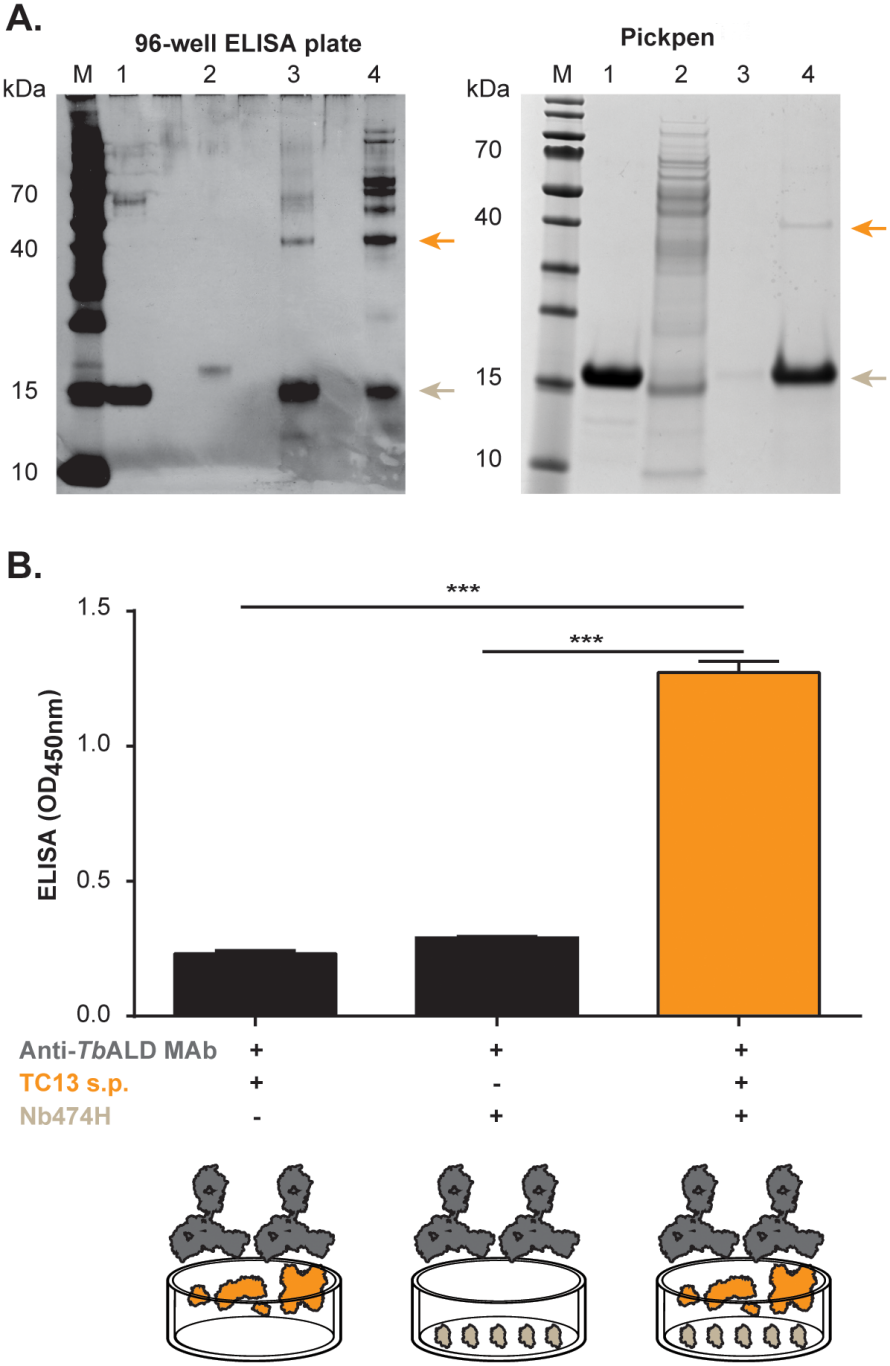
Detection of Nb474H immuno-affinity captured glycosomal aldolase. (A) Nb474H immuno-affinity captured glycosomal aldolase (*Tco*ALD) detected by SDS-PAGE under reducing conditions. Panel (*top left*): Native *Tco*ALD was captured from the soluble proteome (s.p.) or infected sera on a 96-well ELISA coated with Nb474H, eluted from the plate and analysed on a 10% SDS-PAGE developed with silver staining. Lane M, protein ladder; lane 1, pure Nb474H; lane 2, pure Nb474B; lane 3, eluted protein captured from s.p. (6 μg); lane 4, eluted protein captured from infected sera (8 μg). Panel (*top right*): Native *Tco*ALD was captured from secretome on nickel beads linked to Nb474H, eluted and analysed on a 10% SDS-PAGE developed with coomassie blue. Lane M, protein ladder; lane 1, Nb474H; lane 2, flow through; lane 3, wash; lane 4, eluted protein. Native *Tco*ALD protein migrated at ±40 kDa (arrows, top) and the Nb474 migrated at ±15kDa (arrows, bottom). (B) Nb474H immuno-affinity captured *Tco*ALD from *T*. *congolense* TC13 s.p. detected by Anti-*T*. *brucei* aldolase MAb (Anti-*Tb*ALD MAb) in ELISA. Bar (*left*): OD450nm levels in wells filled with coating buffer followed by addition of *T*. *congolense* TC13 s.p. and then Anti-TbALD MAb; bar (*middle*): OD450nm in wells coated with the Nb followed by addition of Anti-*Tb*ALD MAb; and bar (*right*): OD450nm in wells coated with the Nb followed by addition of *T*. *congolense* TC13 s.p. and then Anti-*Tb*ALD MAb. In the last step goat anti-mouse IgG conjugated to Horse radish peroxidase (HRP) was added to all the wells and then developed with 1-Step ultra 3,3′,5,5′-tetramethylbenzidine (TMB) substrate. The OD450nm shown on the graph represent the average value of duplicate wells. *** p<0.001.

Given that the Nb474-ELISA yields a strong positive score when applied to the *T*. *congolense* soluble proteome, the sera of infected mice, or a *T*. *congolense* secretome (S5 Fig), it was reasoned that these samples could be used as the source material from which the target could be purified by Nb474-mediated immuno-capturing. To this end, two distinct approaches were employed. In the first immuno-capturing set-up, Nb474H was adsorbed on 96-well ELISA plate to capture the antigen from the soluble proteome and infected serum. In the second experimental setting, Nb474H was immobilized on nickel beads via its His_6_-tag to capture the antigen from the secretome in an attempt to retrieve the antigen-Nb-bead complex with a Pickpen. The results of both experiments are shown in Fig 6A and 6B, respectively. In both cases, the analysis of the eluted samples via SDS-PAGE reveals a distinct, consistent protein band with an approximate molecular mass of 40 kDa (Fig 6A, lanes 3 and 4; Fig 6B, lane 4). The three bands were analyzed by mass spectrometry to determine the identity of the target. The mass spectrometry results pointed toward *Tco*ALD (UniProtKB accession no. G0UWE7) as the target antigen (S6 Fig). To support the MS findings, a heterologous sandwich ELISA was carried out with Nb474H and Anti-*Tb*ALD as capturing and detecting molecules, respectively. A strong, significant OD_450nm_ signal is observed in the wells coated with Nb474H followed by the subsequent addition of *T*. *congolense* TC13 soluble proteome and the Anti-*Tb*ALD compared to the control wells (Fig 6B).

**Fig 7:**
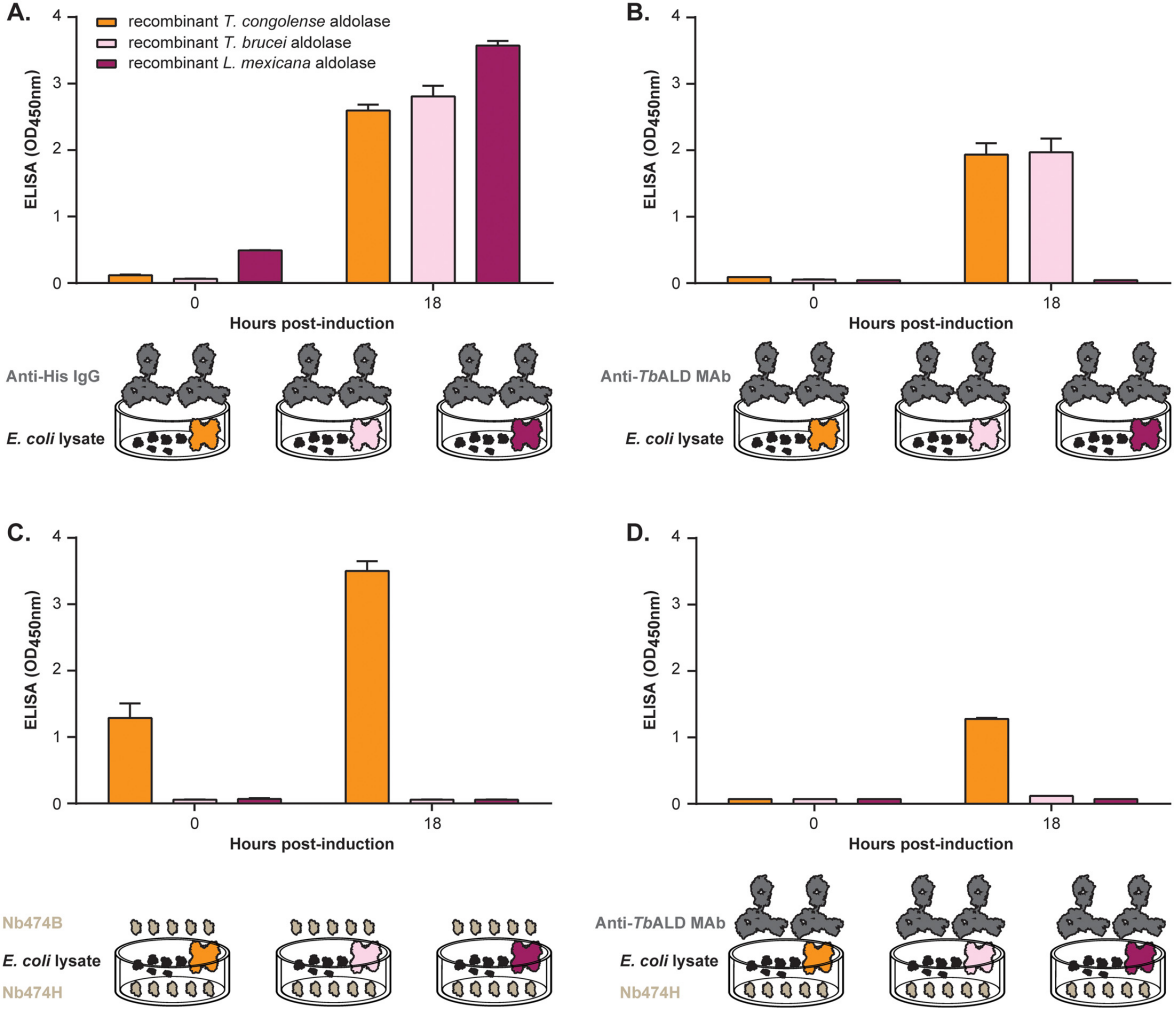
ELISA binding assay performed on recombinant glycosomal aldolase expressed in *E*. *coli*. Crude lysate prepared from cultures obtained before induction of protein expression (0 h) and after induction (18 h) were probed. (A) Anti-His IgG or (B) Anti-*T*. *brucei* aldolase MAb (Anti-*Tb*ALD MAb) was used for probing the presence of expressed aldolase in crude lysate coated on ELISA plate. Anti-His IgG detected all the three recombinant aldolase and Anti-*Tb*ALD MAb only detected trypanosomal aldolase (C) Nb474-based homologous sandwich ELISA (C) or (D) Nb474H-Anti-*Tb*ALD MAb heterologous sandwich ELISA was used to probe the presence of expressed aldolase in crude lysate. Both homologous (Nb474H-Nb474B) and heterologous (Nb474H-Anti-*Tb*ALD MAb) sandwich ELISA detected *T*. *congolense* aldolase only. The OD_450nm_ shown on the graphs represent the average value recorded from the duplicate wells.

Because it was unanticipated that Nb474 could bind *Tco*ALD with such a high level of specificity (given that aldolase is a highly conserved enzyme), *E*. *coli* lysate containing a recombinant version of the enzyme was probed with the Nb474-ELISA. In this validation experiment, lysates prepared from *E*. *coli* producing recombinant *Tb*ALD and *Leishmania mexicana* aldolase (*Lm*ALD) were included as control samples. While from Fig 7A it is evident that all three recombinant proteins are successfully produced in *E*. *coli*, Fig 7B demonstrates that the anti-*Tb*ALD MAb effectively recognizes *Tco*ALD and *Tb*ALD but not *Lm*ALD. The latter can be explained by variation of the linear epitope in *Lm*ALD. In accordance with all of the previous experiments, both the homologous (Nb474H-Nb474B; Fig 7C) and heterologous (Nb474H-Anti-*Tb*ALD MAb; Fig 7D) sandwich ELISAs clearly show that Nb474 binds to *Tco*ALD with high specificity.

## Discussion

The continuous monitoring of infectious diseases is crucial for disease control and treatment. Improved methods for the detection of low levels of pathogen infection and transmission are vital for quick and appropriate responses to prevent any outbreaks. Over the years, immuno-based POC tests have become common diagnostic platform because of their ability to rapidly and reliably detect infections under field conditions. These assays can be formatted in two ways. Detection of infection can be antibody- or antigen-based and both formats have their advantages and drawbacks. Antibody-based diagnosis assays are relatively sensitive as the immune response of the host is used as natural amplification mechanism for pathogen detection. However, a recurring issue with these assays is cross-reactivity, which reduces their specificity. For instance, unrelated diseases associated with polyclonal B-cell/antibody amplification can increase the number of false-positive readings [11]. Furthermore, in some cases host antibodies can circulate in the host long after a pathogen has been cleared thereby yielding a false image of the actual status of the infection. In contrast, antigen-based assays can differentiate between ongoing and past infections [48]. The hurdle in antigen-based detection is the selection of a suitable target antigen. Apart from not cross-reacting in diagnostic test for the detection of other endemic parasites, the target antigen should be present in detectable quantities during an active infection [23,49]. An additional requirement is that the capture and detection antibodies targeting the antigen should be able to out-compete the host anti-pathogen antibodies induced during infection [15,23]. In this paper, we have devised a Nb-based approach that could circumvent the above-mentioned issues hampering the development of antigen-based pathogen detection. Our rationale consists of vaccinating an alpaca with whole-pathogen soluble proteome and applying a stringent panning and screening strategy to deliver the most specific Nbs. While the use of Nb technology allows the selection of specific antigen-binding entities that recognize epitopes different from those bound by conventional antibodies [25], the library generation and screening rationale are performed such as to allow the acquisition of Nbs that recognize unique antigens. Considering that the activity spectrum of diagnostic tests as well as vaccines based on highly immuno-dominant surface antigens of trypanosomes are narrowed by their variant nature, a non-biased antigen source (*T*. *congolense* s.p.) was used to ensure selection of potent Nb(s) recognizing target(s) that are conserved across the different strains of *T*. *congolense*.

To test our strategy, we employed *T*. *congolense* as a case study. The application of our approach led to the identification of a potent *T*. *congolense* specific Nb, Nb474. By making His-tagged and biotinylated versions of this Nb (Nb474H and Nb474B, respectively), we were able to construct a homologous sandwich ELISA that was tested for its diagnostic potential in different scenarios. In an experimental mouse model, the assay is able to detect infections with different *T*. *congolense* strains, although there is a significant variation in the measured O.D. values. The observed variation is attributed to uneven parasite densities at the time point when samples were collected. Overall, the fact that all the strains tested show O.D. readings significantly above those of the negative samples or those taken from mice that were infected with trypanosomes other than *T*. *congolense* is an indication of the assay’s specificity for the diagnosis of *T*. *congolense* infections.

The *T*. *congolense* infected mice display a typical parasitemia profile, which is characterized by a parasitemia peak around day 7 post infection. This first parasite wave rapidly drops due to the rapid destruction of trypanosomes by the host (commonly known as ‘trypanolytic crisis’), after which the parasitemia drops below the detection limit of parasitological techniques. Indeed, in accordance with the literature [48,50], the blood parasite load was very scanty by microscopy after the parasite peak despite the presence of trypanosomes in the circulation. Interestingly, the antigenemia detected via the Nb474-ELISA remains significantly high, even after the first parasite wave. Upon clearance of the parasites from the system, the signal of the Nb474-ELISA is reduced to zero, suggesting that the occurrence of the detected antigen in the circulation is linked to the presence of parasites in the host and that the antigen does not persist after parasite clearance. Hence, in experimental mice infections, the Nb474-ELISA has the ability to distinguish active infections from a cured state and can thus be employed as a test-of-cure. By being able to differentiate sick animals from cured, the assay might in part qualify as the first-line of test for drug resistance. This feature of the ELISA, from the clinician’s perspective, is useful for monitoring the efficacy of trypanocidal drugs.

The Nb474-ELISA was also able to diagnose *T*. *congolense* infections in experimentally and naturally infected cattle. The obtained results showed potential application of the diagnostic tool in the laboratory settings. Additionally, it seems that delayed sample investigation does not affect the assay’s sensitivity provided that the collected samples are kept under cold storage conditions. In the endemic areas, delayed sample investigation is often experienced in case of electricity failure or when there are no efficient means for sample delivery to distant laboratories. Together with its current level of performance, the option of delayed sample investigation would make this assay a suitable tool for monitoring *T*. *congolense* infections in remote settings and retrospective analysis of archived samples. We plan to further validate this assay on larger collections of field samples from different geographical locations where the disease is endemic.

Finally, we identified the target antigen of the Nb474-ELISA as being *Tco*ALD, which is a glycolytic enzyme found in the glycosomes [51]. Interestingly, *Tco*ALD has been reported to be a part of the so-called *T*. *congolense* secretome [52]. However, the exact mechanism by which the enzyme reaches the host’s circulation is yet to be resolved. The findings in this paper demonstrate that *Tco*ALD is a suitable biomarker for the identification of *T*. *congolense* infections, despite its high sequence identity with the glycosomal aldolase of other parasites (S1 Table). This is not the first report presenting aldolase as a specific biomarker for pathogen detection. The enzyme variant encoded by *Plasmodium vivax* serves as a biomarker for the diagnosis of malaria [53]. Based on the structures of aldolases encoded by *T*. *brucei* and *L*. *mexicana* [54], *Toxoplasma gondii* [55] and *P*. *falciparum* [56], it is expected that *Tco*ALD is a homo-tetramer in solution. This also explains why the Nb474 sandwich ELISA can be used in a homologous set-up. In the case of a monomeric target antigen, the same Nb could evidently not be used as a capturing and a detection reagent because the antigen would harbour only a single binding site for the Nb, which necessitates the development of a heterologous sandwich ELISA. However, the principle of a homologous sandwich ELISA is applicable for an oligomeric target antigen [57]. The observation that Nb474 can be used for antigen capturing as well as detection suggests the occurrence of at least two Nb474-binding sites on *Tco*ALD. However, this remains speculative as the structural and biophysical determinants of the specific Nb474-*Tco*ALD interaction remain yet to be investigated. We expect that these studies will help us improve the robustness of the Nb474-ELISA to specifically monitor *T*. *congolense* infections in the field. Moreover, the advantage of a selective test for trypanosomosis in the regions of sub-Saharan Africa where animals are infected with human as well as animal infective trypanosomes is that it discriminates between the two parasite groups, hence enabling assessment of the potential risk for human infection [49]. Further development of the ELISA will bring us a step closer towards obtaining an antigen-detection immuno-based POC test that can be used for rapid and reliable detection of pathogen infections.

## Supporting Information

S1 Materials and Methods Titration of Capturing Nb (Nb474H) against Detecting Nb (Nb474B). (PDF)

S1 Fig Detection of *E*. *coli* colonies expressing cross-reactive Nbs binding mouse serum protein. (TIF)

S2 Fig Translated protein sequence of anti-*T*. *congolense* glycosomal aldolase Nanobody (Nb474). Frame work regions (FR 1–3) and complementarity determining regions (CDRs) are demarcated according to the international Immunogenetics information system of numbering (http://igmt.cines.fr). The color code blue, green and orange represents CDR1, CDR2 and CDR3, respectively.
(TIF)

S3 Fig Detection limit of Nb474-ELISA. (TIF)

S4 Fig Sensitivity of Nb474-ELISA on infected serum stored at different temperatures for specified time periods. (TIF)

S5 Fig Nb474-ELISA recognized *T*. *congolense* infected mouse serum, soluble protein and secretome. (A) Pooled naive and positive mice sera were tested alongside TC13 soluble proteome (s.p.). (B) Pooled naive and positive mice sera were tested alongside *T*. *congolense* IL3000 secretome. A high OD_450nm_ was observed on *T*. *congolense* positive serum, soluble proteome and secretome suggesting occurrence of a common antigen in all the three sample types. The OD_450nm_ shown on the graphs represents the average value of the duplicate wells. n.s. = non-significant, ** p<0.01, *** p<0.001.
(TIF)

S6 Fig Mass spectrometry (MS) detected peptides that matched the shaded regions on the sequence of *T*. *congolense* glycosomal aldolase. MS analysis recovered several peptides covering up to 36.29% of the entire *Tco*ALD sequence.
(TIF)

S1 Table Identity matrix showing the similarity in the sequence of *T*. *congolense* glycosomal aldolase to that of other trypanosomes, *L*. *mexicana* and cattle. (PDF)
