# Supplementary material for: An Anti-proteome Nanobody Library Approach Yields a Specific Immunoassay for Trypanosoma congolense Diagnosis Targeting Glycosomal Aldolase
Source: PLoS Negl Trop Dis. 2016 Feb 2;10(2):e0004420. doi: 10.1371/journal.pntd.0004420 (PMC4737498; doi:10.1371/journal.pntd.0004420)
Supplement: S1 Materials and Methods — (PDF) [file pntd.0004420.s001.pdf]

S1 Materials and Methods. Titration of Capturing Nb (Nb474H) against Detecting Nb (Nb474B).

Nb474H (1.26 µg/ml) was serially diluted (2-fold) in PBS starting from (0.63-0.01) µg/ml. The dilutions were coated row-wise (from wells in row A-G) in a decreasing concentration. For example, wells in row A and G received 0.63 and 0.01 µg/ml, respectively. The control wells (row H) received coating buffer only. Uncoated Nb474H was washed with PBS containing 0.05% Tween20 (PBS-T) three times and wells were blocked with 300 µl 5% (w/v) milk for 2 h at 22°C. Blocking buffer was washed three times and *T. congolense* s.p. was added (0.16 µg/well). Incubation was allowed for 1 h at 22 °C and washed three times. Nb474B (1.26 µg/ml) was serially diluted in blocking buffer (2-fold) from 0.63-0.0006 µg/ml. The dilutions were added column-wise (from wells in column 1-11) in a decreasing concentration. For example, wells in column 1 and 11 received 0.63 µg/ml and 0.0006 µg/ml Nb474B, respectively. Wells in column 12 received blocking buffer only. Incubation was allowed for 1 h at 22 °C and then washed four times. Strep-HRP diluted (1µg/ml) in blocking buffer was added followed by an hour of incubation. Wells were washed off unbound conjugates five times. The retention of Strep-HRP was reported by adding TMB substrate. The reaction was allowed for 25 min, stopped with 1M sulphuric acid and the absorbance was read.
