# Supplementary figures and images for: An Anti-proteome Nanobody Library Approach Yields a Specific Immunoassay for Trypanosoma congolense Diagnosis Targeting Glycosomal Aldolase
Source: PLoS Negl Trop Dis. 2016 Feb 2;10(2):e0004420. doi: 10.1371/journal.pntd.0004420 (PMC4737498; doi:10.1371/journal.pntd.0004420)

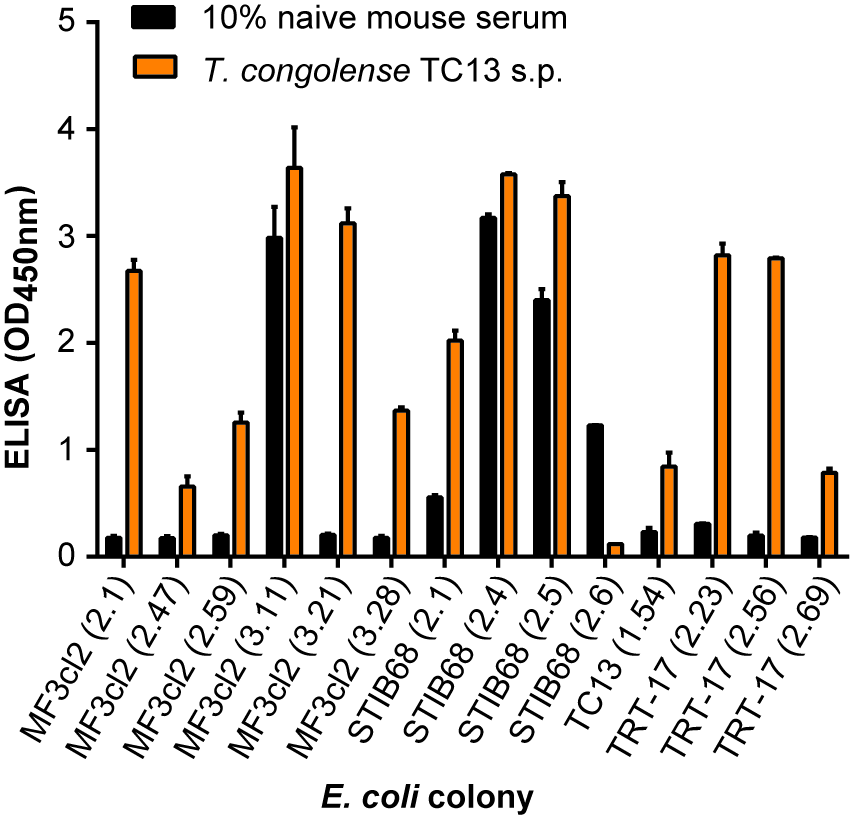

Supplement: S1 Fig — (TIF) [file pntd.0004420.s002.tif]

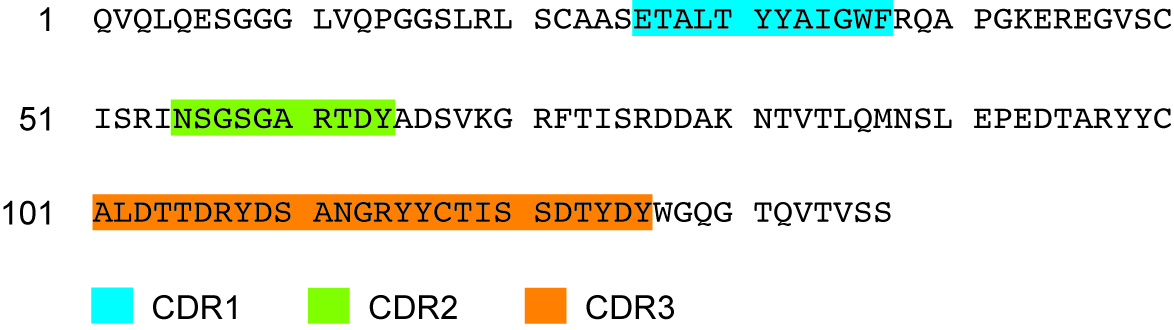

Supplement: S2 Fig — Frame work regions (FR 1–3) and complementarity determining regions (CDRs) are demarcated according to the international Immunogenetics information system of numbering (http://igmt.cines.fr). The color code blue, green and orange represents CDR1, CDR2 and CDR3, respectively. (TIF) [file pntd.0004420.s003.tif]

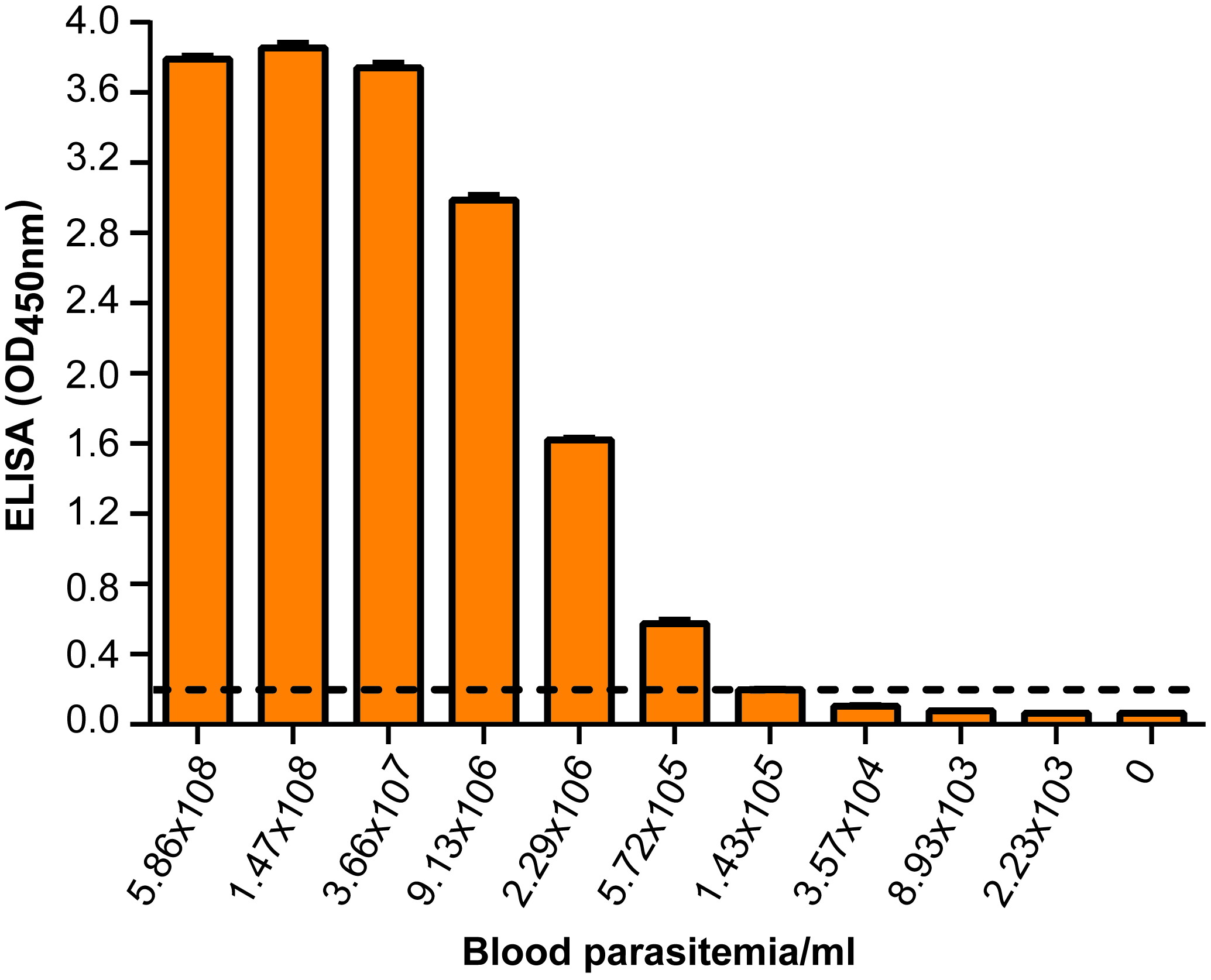

Supplement: S3 Fig — (TIF) [file pntd.0004420.s004.tif]

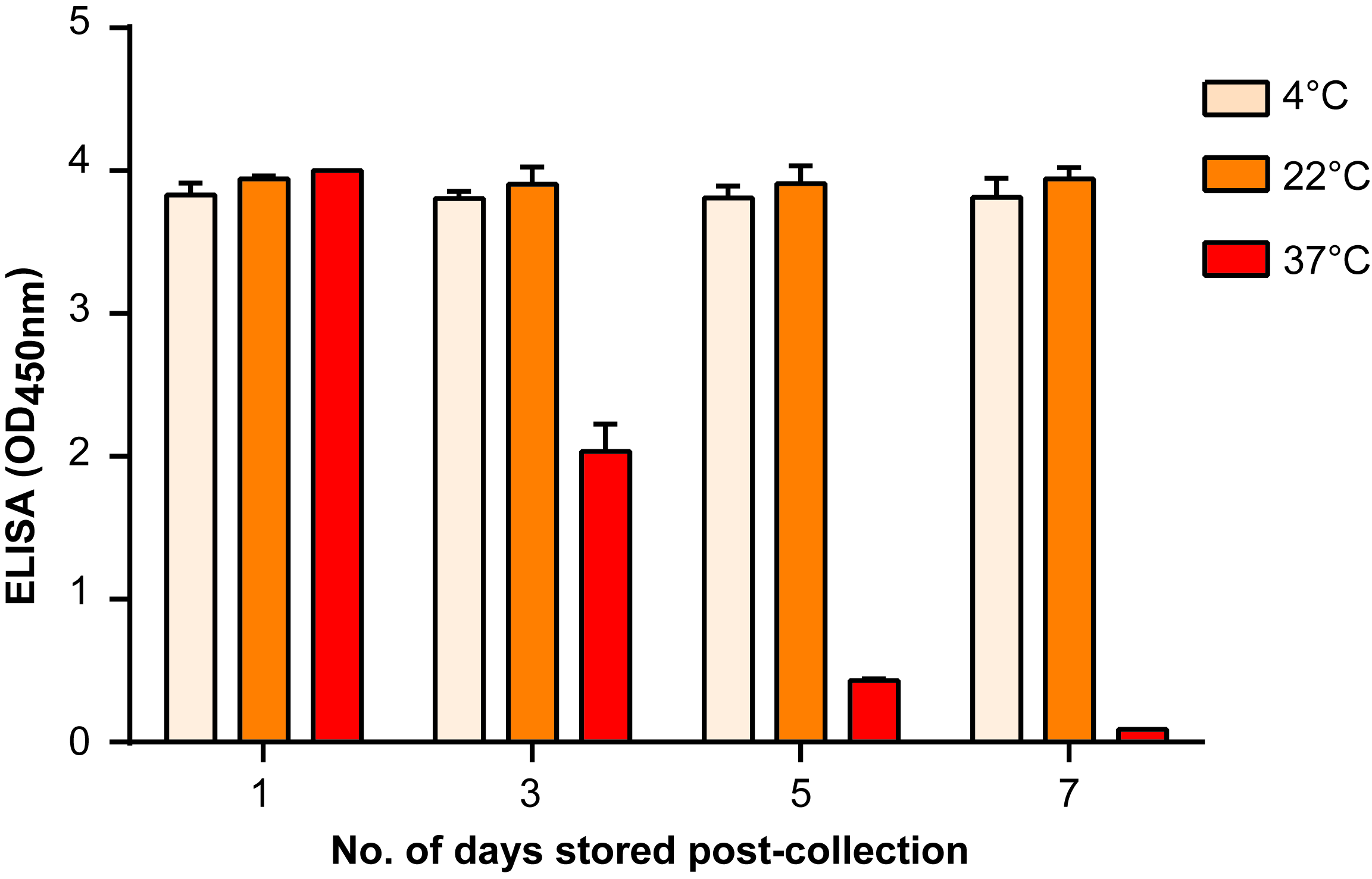

Supplement: S4 Fig — (TIF) [file pntd.0004420.s005.tif]

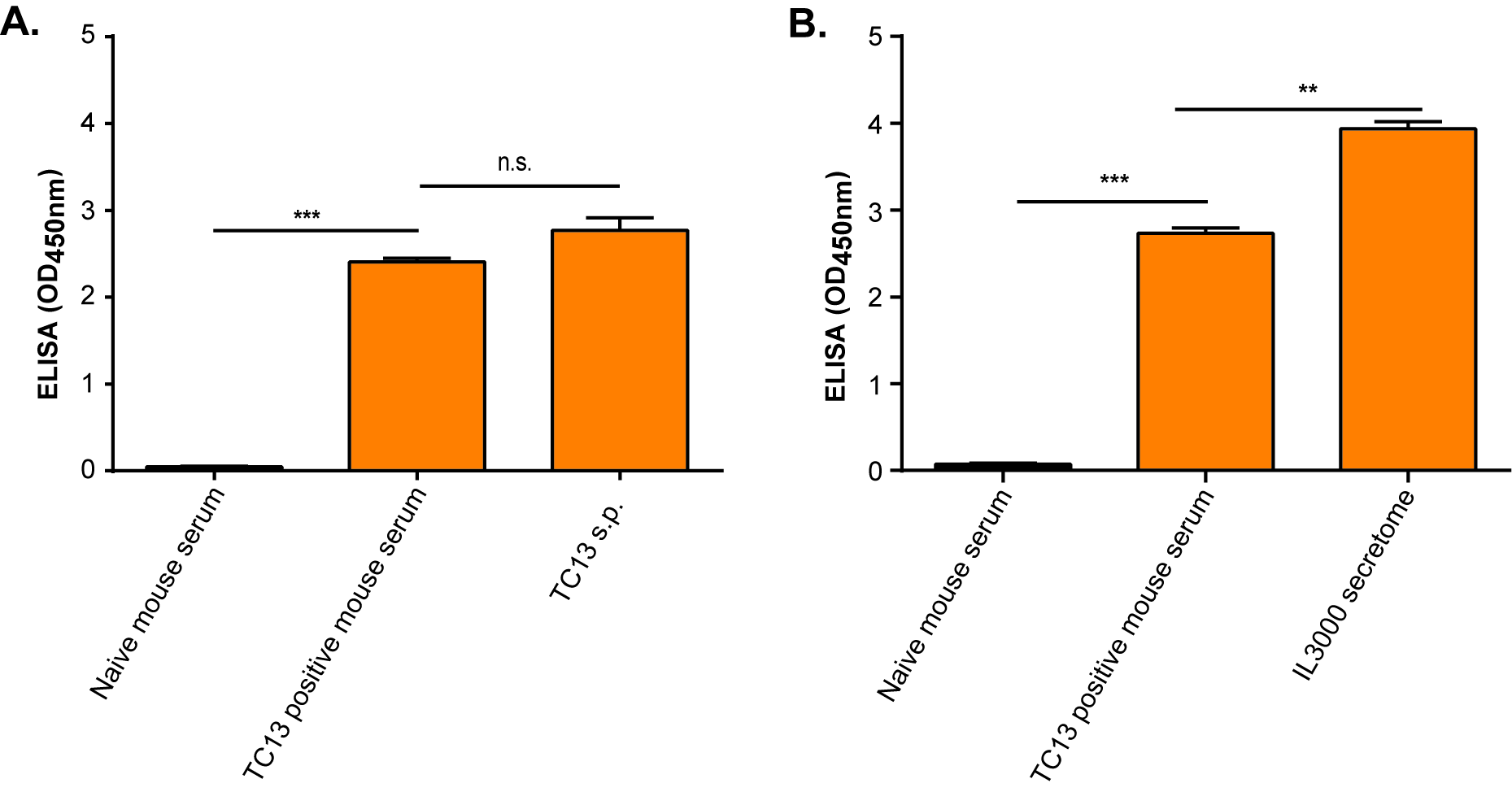

Supplement: S5 Fig — (A) Pooled naive and positive mice sera were tested alongside TC13 soluble proteome (s.p.). (B) Pooled naive and positive mice sera were tested alongside T. congolense IL3000 secretome. A high OD450nm was observed on T. congolense positive serum, soluble proteome and secretome suggesting occurrence of a common antigen in all the three sample types. The OD450nm shown on the graphs represents the average value of the duplicate wells. n.s. = non-significant, ** p<0.01, *** p<0.001. (TIF) [file pntd.0004420.s006.tif]

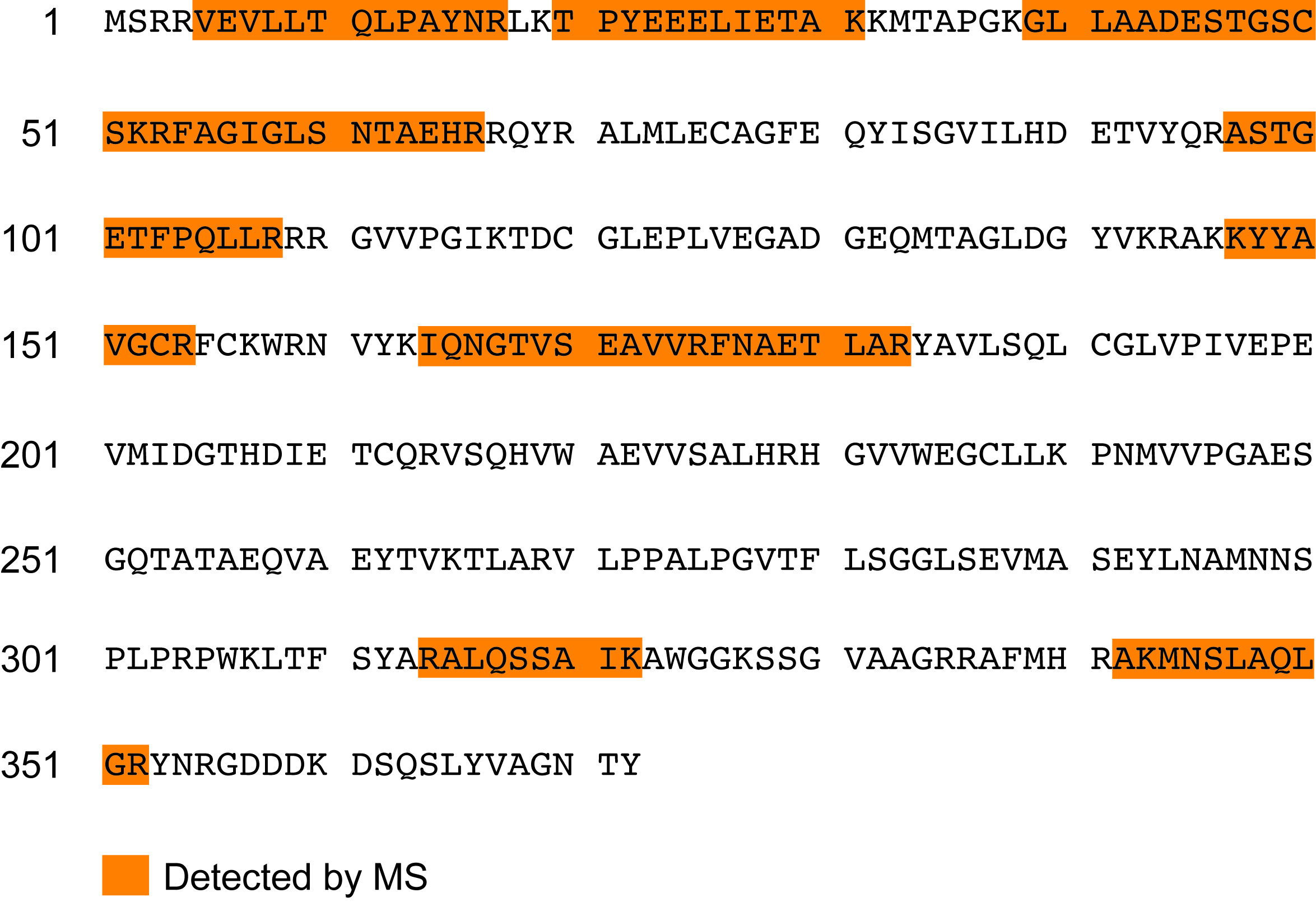

Supplement: S6 Fig — MS analysis recovered several peptides covering up to 36.29% of the entire TcoALD sequence. (TIF) [file pntd.0004420.s007.tif]
