# Supplementary material for: An Anti-proteome Nanobody Library Approach Yields a Specific Immunoassay for Trypanosoma congolense Diagnosis Targeting Glycosomal Aldolase
Source: PLoS Negl Trop Dis. 2016 Feb 2;10(2):e0004420. doi: 10.1371/journal.pntd.0004420 (PMC4737498; doi:10.1371/journal.pntd.0004420)
Supplement: S1 Table — (PDF) [file pntd.0004420.s008.pdf]

S1 Table. Identity matrix showing the similarity in the sequence of *T. congolense* glycosomal aldolase to that of other trypanosomes, *L. mexicana* and cattle.

	Q3T0S5	G0U6L2	G0UWE7	P07752	TevSTIB805[a] .10.5960	K4DLE5	Q9U5N6
Q3T0S5		45.1	46.9	46.9	47.5	47.5	47.7
G0U6L2	45.1		85.2	86.0	86.6	84.9	80.9
G0UWE7	46.9	85.2		94.1	94.9	87.9	80.1
P07752	46.9	86.0	94.1		99.2	86.3	79.8
TevSTIB805[a] .10.5960	47.5	86.6	94.9	99.2		87.1	80.4
K4DLE5	47.5	84.9	87.9	86.3	87.1		79.8
Q9U5N6	47.7	80.9	80.1	79.8	80.4	79.8	

T. congolense (UniProtKB accession no. G0UWE7), cattle (UniProtKB accession no. Q3T0S5), *T. vivax* (UniProtKB accession no. G0U6L2), *T. brucei* (UniProtKB accession no. P07752), *T. evansi* (TriTrypDB accession no. TevSTIB805.10.5960), *T. cruzi* (UniProtKB accession no. K4LE5) and *L. mexicana* (UniProtKB accession no. Q9U5N6).

[a] TriTrypDB accession no. TevSTIB805.10.5960.
